# Ultrasound-assisted carbon nanoparticle suspension mapping *versus* dual tracer-guided sentinel lymph node biopsy in patients with early breast cancer (ultraCars): phase III randomized clinical trial

**DOI:** 10.1093/bjs/znac311

**Published:** 2022-09-08

**Authors:** Liulu Zhang, Minyi Cheng, Yingyi Lin, Junsheng Zhang, Bo Shen, Yuanqi Chen, Ciqiu Yang, Mei Yang, Teng Zhu, Hongfei Gao, Fei Ji, Jieqing Li, Kun Wang

**Affiliations:** Department of Breast Cancer, Cancer Centre, Guangdong Provincial People’s Hospital, Guangdong Academy of Medical Sciences, Guangzhou, China; Department of Breast Cancer, Cancer Centre, Guangdong Provincial People’s Hospital, Guangdong Academy of Medical Sciences, Guangzhou, China; Second School of Clinical Medicine, Southern Medical University, Guangzhou, China; Department of Breast Cancer, Cancer Centre, Guangdong Provincial People’s Hospital, Guangdong Academy of Medical Sciences, Guangzhou, China; Shantou University Medical College, Shantou, Guangdong, China; Department of Breast Cancer, Cancer Centre, Guangdong Provincial People’s Hospital, Guangdong Academy of Medical Sciences, Guangzhou, China; Shantou University Medical College, Shantou, Guangdong, China; Department of Breast Cancer, Cancer Centre, Guangdong Provincial People’s Hospital, Guangdong Academy of Medical Sciences, Guangzhou, China; Shantou University Medical College, Shantou, Guangdong, China; Department of Breast Cancer, Cancer Centre, Guangdong Provincial People’s Hospital, Guangdong Academy of Medical Sciences, Guangzhou, China; Second School of Clinical Medicine, Southern Medical University, Guangzhou, China; Department of Breast Cancer, Cancer Centre, Guangdong Provincial People’s Hospital, Guangdong Academy of Medical Sciences, Guangzhou, China; Department of Breast Cancer, Cancer Centre, Guangdong Provincial People’s Hospital, Guangdong Academy of Medical Sciences, Guangzhou, China; Department of Breast Cancer, Cancer Centre, Guangdong Provincial People’s Hospital, Guangdong Academy of Medical Sciences, Guangzhou, China; Department of Breast Cancer, Cancer Centre, Guangdong Provincial People’s Hospital, Guangdong Academy of Medical Sciences, Guangzhou, China; Department of Breast Cancer, Cancer Centre, Guangdong Provincial People’s Hospital, Guangdong Academy of Medical Sciences, Guangzhou, China; Department of Breast Cancer, Cancer Centre, Guangdong Provincial People’s Hospital, Guangdong Academy of Medical Sciences, Guangzhou, China; Department of Breast Cancer, Cancer Centre, Guangdong Provincial People’s Hospital, Guangdong Academy of Medical Sciences, Guangzhou, China; Second School of Clinical Medicine, Southern Medical University, Guangzhou, China

## Abstract

**Background:**

Appropriate tracing methods for sentinel lymph node biopsy (SLNB) play a key role in accurate axillary staging. This prospective, non-inferiority, phase III RCT compared the feasibility and diagnostic performance of ultrasound-assisted carbon nanoparticle suspension (CNS) mapping with dual tracer-guided SLNB in patients with early breast cancer.

**Methods:**

Eligible patients had primary breast cancer without nodal involvement (cN0), or had clinically positive lymph nodes (cN1) that were downstaged to cN0 after neoadjuvant chemotherapy. Patients were randomly assigned (1 : 1) to undergo either ultrasound-assisted CNS sentinel lymph node (SLN) mapping (UC group) or dual tracer-guided mapping with CNS plus indocyanine green (ICG) (GC group). The primary endpoint was the SLN identification rate.

**Results:**

Between 1 December 2019 and 30 April 2021, 330 patients were assigned randomly to the UC (163 patients) or GC (167 patients) group. The SLN identification rate was 94.5 (95 per cent c.i. 90.9 to 98.0) per cent in the UC group and 95.8 (92.7 to 98.9) per cent in the GC group. The observed difference of –1.3 (–5.9 to 3.3) per cent was lower than the prespecified non-inferiority margin of 6 per cent (P_non–inferiority_ = 0.024). No significant difference was observed in metastatic node rate (30.5 *versus* 24.4 per cent; *P* = 0.222), median number of SLNs harvested (3 (range 1–7) *versus* 3 (1–8); *P* = 0.181), or duration of surgery (mean(s.d.) 7.53(2.77) *versus* 7.63(3.27) min; *P* = 0.316) between the groups. Among the subgroup of patients who had undergone neoadjuvant treatment, the SLN identification rate was 91.7 (82.2 to 100) per cent in the UC group and 90.7 (81.7 to 99.7) per cent in the GC group.

**Conclusion:**

The diagnostic performance of ultrasound-assisted CNS mapping was non-inferior to that of dual tracer-guided SLN mapping with CNS plus ICG in patients with early breast cancer.

**Registration number:**

NCT04951245 (http://www.clinicaltrials.gov).

## Teaser

The diagnostic performance of ultrasound-assisted carbon nanoparticle suspension (CNS) sentinel lymph node (SLN) mapping was non-inferior to that of dual tracer-guided mapping in patients with early breast cancer. Ultrasound-assisted CNS mapping also offers additional potential clinical utility for SLN biopsy (SLNB) after neoadjuvant chemotherapy. This study suggests that ultrasound-assisted CNS mapping has great value for clinical application and could serve as a useful alternative to dual tracer-guided SLNB.

## Introduction

Ascertainment of regional lymph node status in breast cancer is essential for local control, stage determination, and estimating prognosis. Sentinel lymph node biopsy (SLNB) is the standard procedure for axillary staging in patients with early breast cancer without clinical or radiological evidence of lymph node metastasis^1–5^. SLNB can also be performed in patients who achieve clinical axillary lymph node conversion after preoperative systemic treatment, thus potentially sparing them from axillary lymph node dissection (ALND)^6–9^.

Appropriate tracing methods for SLNB play a key role in axillary staging. The current standard for detection of the sentinel lymph node (SLN) is the dual tracer-guided technique comprising use of radioisotope and blue dye; this has an identification rate above 90 per cent and false-negative rate lower than 10 per cent^1,2,10,11^. Radioisotope availability is, however, restricted in some countries owing to complex legislation and challenges in managing radioactive substances. This has led to the development of alternative methods for SLN mapping, such as use of indocyanine green (ICG). Near-infrared (NIR) fluorescence imaging with ICG can achieve clear visualization of lymphatic vessels and nodes. The identification and false-negative rates of ICG combined with blue dye are comparable to those of radioisotope combined with blue dye, and there is increasing evidence to support the use of ICG and blue dye in SLN mapping^12^. Application is hindered by the requirement for an NIR camera and the potential complications associated with blue dye injection, including anaphylactic reaction and local skin inflammation or necrosis^13,14^.

Carbon nanoparticle suspension (CNS) can easily penetrate lymphatic vessels while seldom entering the blood circulation because particles have a diameter of 150 nm. A previous observational study^15^ showed that CNS-based SLNB had an identification rate of 99.1 per cent (329 of 332) and a false-negative rate of 4.1 per cent (2 of 49), with few adverse events reported. The 2021 Chinese Society of Breast Surgery practice guidelines^16^ consider CNS as a promising SLN tracer that could be applied more broadly in clinical practice. Nevertheless, localizing SLNs with CNSs only largely relies on naked-eye observation and personal experience, which may prolong the operation and reduce the accuracy of SLN identification.

In recent years, with the development of high-frequency ultrasound imaging, ultrasound-guided surgery has become an appealing option for breast surgeons given the high sensitivity, non-invasiveness, and portability of intraoperative ultrasound systems, with the additional benefits of accurate surgical margin assessment, lower reoperation rates, and normal tissue preservation in breast-conserving surgery^17^.

Ultrasound-assisted CNS mapping might be of clinical value and serve as a useful alternative to dual tracer-guided SLNB. This prospective phase III RCT was designed to compare the feasibility and diagnostic performance of ultrasound-assisted CNS mapping with dual tracer-guided SLNB in patients with early breast cancer.

## Methods

### Study design and patients

This was single-centre, open-label, non-inferiority, phase III RCT. Patients were recruited between 1 December 2019 and 30 April 2021 at Guangdong Provincial People’s Hospital, Guangdong Academy of Medical Sciences (Guangzhou, China). Eligible patients were aged 18 year­s or older, and had either histologically confirmed primary invasive breast cancer or ductal carcinoma *in situ* scheduled for mastectomy. Patients had no clinical or radiological nodal involvement (cN0), or had clinically positive lymph nodes (cN1) downstaged to cN0 following neoadjuvant chemotherapy (NACT), and an Eastern Cooperative Oncology Group performance status score of 0 or 1. Axillary involvement was defined following the seventh edition of the AJCC staging system. Patients were ineligible if they had T4 tumours, had previously undergone axillary radiotherapy or surgery, or were pregnant or breastfeeding.

The study was approved by the Research Ethics Committee (GDREC2019610H) and conducted in accordance with the Declaration of Helsinki, guidelines for Good Clinical Practice, and the CONSORT statement. All patients provided written informed consent. The trial was registered at ClinicalTrials.gov (NCT04951245).

### Randomization and masking

An interactive response system was used to obtain treatment assignments. Patients were assigned randomly in a 1 : 1 ratio, using a permuted block randomization scheme, to undergo either ultrasound-assisted CNS SLN mapping (UC group) or dual tracer-guided mapping with CNS plus ICG (GC group). The study was unmasked, and patients, investigators and surgeons were all aware of the study group assignment.

### Procedures

Before this trial, four individual surgeons participated in training supervised by a breast radiologist and an experienced surgeon. Each surgeon completed 20 procedures separately to implement intraoperative ultrasound-assisted SLNB. The learning curve for the procedure is shown in *Fig. S1*.

Primary tumour and nodal status were assessed by physical examination, ultrasound imaging, mammography, and MRI within 2 weeks of surgery. For patients undergoing NACT, surgery was performed between 4 and 8 weeks after the last dose of chemotherapy. According to institutional practice, ultrasound-guided core-needle biopsy of suspicious axillary lymph nodes was carried out before the administration of NACT, and a clip marker was placed within the sampled lymph node. A node considered suspicious based on ultrasound characteristics is characterized by eccentric cortical enlargement (over 3 mm) or lobulation with displacement of the hilum, absent hilum or irregular borders, and spherical shape. Quality control of SLNB after NACT entailed removal of the clip-containing node and at least three lymph nodes, with use of intraoperative radiography to identify the clipped node. The choice of primary tumour procedure was decided before operation based on clinical grounds and performed after SLNB had been completed.

**Fig. 1:**
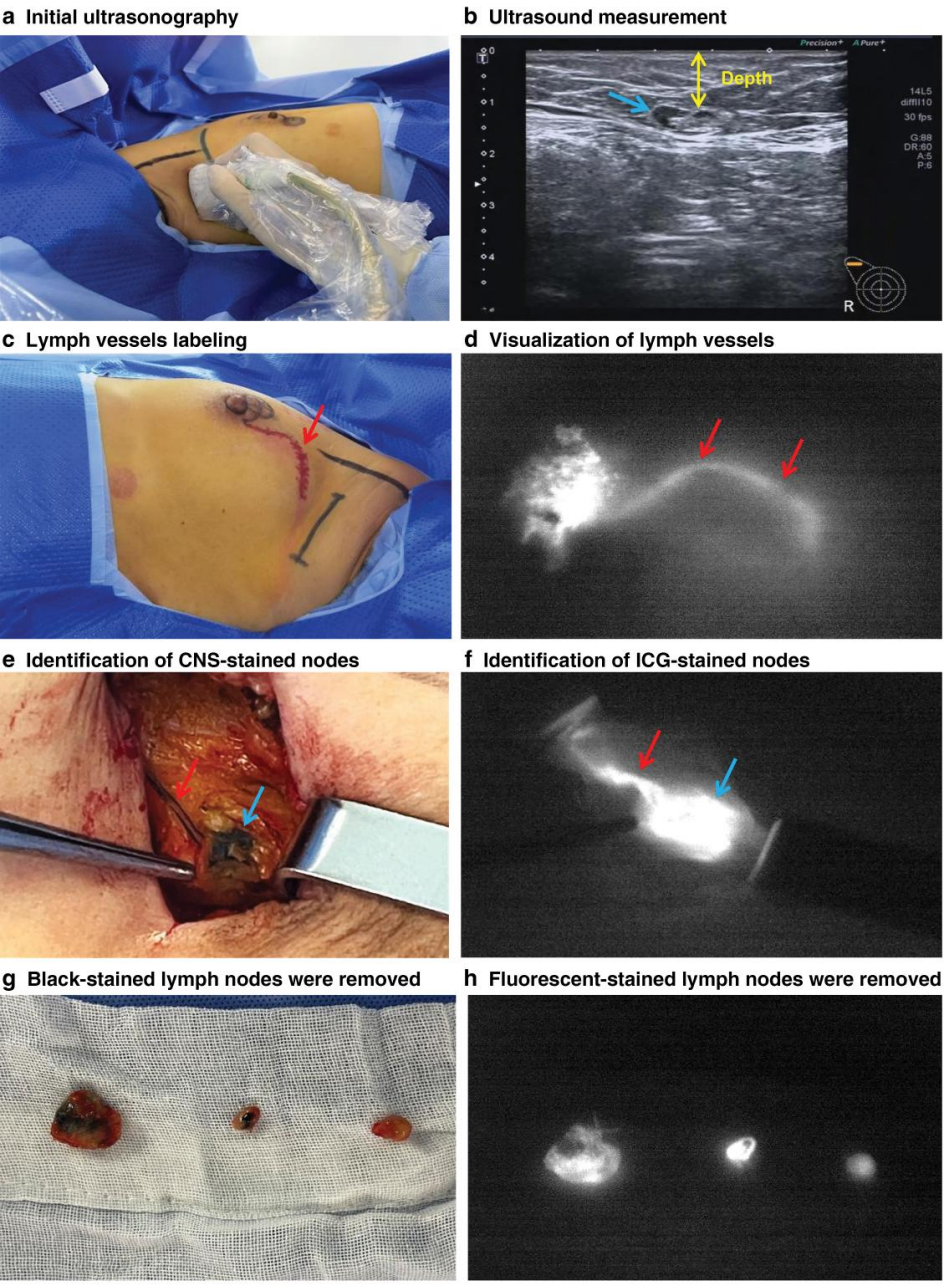
Sentinel lymph node biopsy procedures **a** Ultrasound-guided exploration of sentinel lymph nodes (SLNs) before skin incision. **b** Distance from skin to nodes (arrow) was measured by ultrasound imaging and recorded in millimetres. **c–f** Visualization of subcutaneous lymph vessels (arrow) and localization of SLNs (arrow). **e** Identification of carbon nanoparticle suspension-stained nodes. **g**,**h** Fluorescent or black-stained lymph nodes were removed.

In the UC group, 1 ml CNS (China Food and Drug Administration approval H20041829; Lai Mei Pharmaceutical Company™, Chongqing, China) was injected subcutaneously into the areolar area in the upper outer quadrant of the breast. The injection site was massaged for 15 min to promote drainage of the tracer to the axilla. During the intraoperative ultrasound-assisted procedure, an ultrasound diagnostic system (TOSHIBA APLIO 400, Japan) was used. Before making the incision, ultrasound-guided exploration of the SLNs was undertaken by placing the probe on the lateral border of the breast and sliding cranially along the lateral border of the pectoralis major muscle (*Fig. 1a*). SLNs were usually located in the area adjacent to the lateral thoracic tributary of the axillary vein (LTV), extending from the lower border of the axilla to the second intercostobrachial nerve. Axillary ultrasonography was performed to identify the lateral thoracic vein, and the vast majority of patients had lymph nodes in this predetermined anatomical region. A sterile skin marker was used to mark the optimal site of incision over the targeted lymph nodes, and the distance from the skin to the nodes was measured by ultrasound imaging and recorded in millimetres (*Fig. 1b*). Blunt dissection was carried out to identify the CNS-stained nodes around the marked region. An ultrasonography probe was placed repeatedly in or around the wound at different angles for adequate visualization if SLNs could not be localized with further dissection. All black-stained lymph nodes or palpable suspicious lymph nodes were excised (*Fig. 1e*,*g*).

In the GC group, the preparation for CNS mapping was identical to that in the UC group; 1 ml diluted ICG (2.5 mg/ml; Medical Pharmaceutical Company) was subsequently injected into the areolar area in the upper outer quadrant of the breast. An NIR camera (Dipu Medical Technology Company) was used to visualize the subcutaneous lymph vessels and localize the SLNs (*Fig. 1c*,*d*,*f*). All fluorescent or black-stained lymph nodes were removed along with any palpable suspicious nodes (*Fig. 1g*,*h*). The remaining surgical field was re-examined to ensure complete resection of fluorescent lymph nodes.

All removed lymph nodes were sent for intraoperative frozen-section analysis and subsequent pathological evaluation. Paraffin-embedded sections were deparaffinized and stained with haematoxylin and eosin, and immunohistochemical staining was used to confirm suspected metastasis. ALND was undertaken according to the histological findings of the SLNs and current international guidelines.

### Endpoints

The primary endpoint was the identification rate of SLNs, calculated as the number of patients in whom at least one SLN was detected divided by the total number of patients enrolled. Secondary endpoints included the median number of SLNs collected; the metastasis rate of SLNs, calculated as the number of patients who had at least one positive node divided by the number of patients whose SLNs were identified; duration of surgery, defined as the time from skin incision to resection of the SLN specimens in patients in whom at least one sentinel node was detected; and intraoperative or postoperative complications. The identification rate of SLNs in patients who had undergone NACT was investigated in an exploratory analysis.

### Statistical analysis

PASS 2019 software was used to calculate the sample size. A non-inferiority (1-sided) hypothesis was adopted, which assumed an SLN identification rate of 96 per cent for each group and a non-inferiority margin (Δ) of 6 per cent to secure an identification rate above 90 per cent (1-sided test significance level (α) = 0.05)^12^. A sample size of 264 patients was required to achieve 80 per cent power to reject the null hypothesis that the identification rate in the UC group was inferior to that in the GC group by more than a 6 per cent non-inferiority margin (with a 5 per cent probability of type I error). Anticipating a 10 per cent drop-out rate, at least 294 patients would need to be recruited.

The results are presented as the mean ± standard deviation (SD) or median (range). To compare the intervention and control groups, the χ^2^ test or Fisher’s exact test was used for analysis of categorical variables and the *t* test for continuous variables. *P* < 0.050 signified statistical significance. The intention-to-treat method was used and the analysis included all patients who underwent SLNB. SPSS^®^ software version 26 (IBM, Armonk, NY, USA) was used for statistical analyses.

## Results

**Fig. 2:**
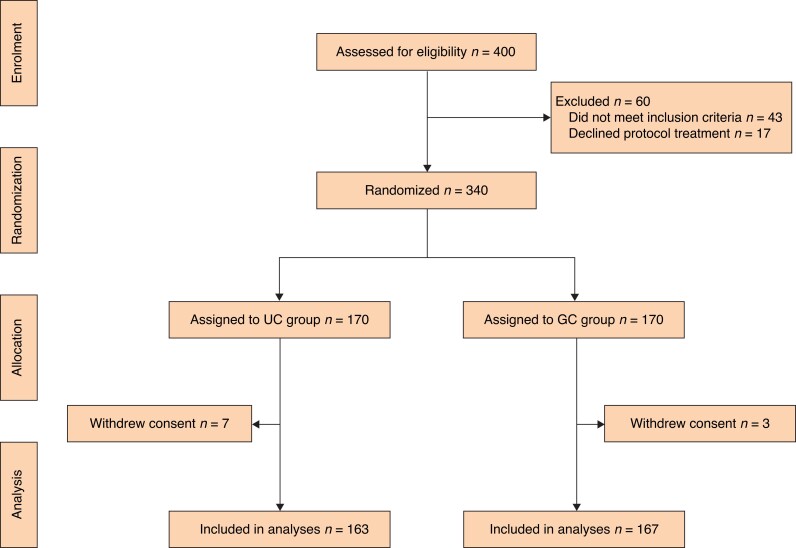
Study flow diagram UC group, ultrasound-assisted carbon nanoparticle suspension (CNS) sentinel lymph node (SLN) mapping; CG group, dual tracer-guided SLN mapping using CNS plus indocyanine green.

Between 1 December 2019 and 30 April 2021, 400 patients were screened, of whom 340 met the enrolment criteria. Ten individuals were excluded owing to withdrawal of informed consent (*Fig. 2*). The remaining 330 patients, assigned randomly to the UC group (163 patients) or the GC group (167 patients), were included in the primary endpoint analysis.

Clinical and pathological characteristics were comparable between the two groups (*Table 1*). Median age was 48 (range 23–74) years. Overall, 79 patients (23.9 per cent) underwent NACT, and 49 (14.8 per cent) had clinically positive axillary lymph nodes (cN1) at initial evaluation. In total, 233 patients (70.6 per cent) underwent SLNB only and 97 (29.4 per cent) had a subsequent ALND.

**Table 1:** Patient characteristics

	Total (*n* = 330)	UC group (*n* = 163)	GC group (*n* = 167)
**Age (years), median (range)**	48 (23–74)	49 (26–74)	47 (23–70)
**Clinical T category at diagnosis**			
cTis	36 (10.9)	21 (12.9)	15 (9.0)
cT1	121 (36.7)	68 (41.7)	53 (31.7)
cT2	154 (46.6)	65 (39.9)	89 (53.3)
cT3	19 (5.8)	9 (5.5)	10 (6.0)
**Clinical N category at diagnosis**			
cN0	281 (85.2)	139 (85.3)	142 (85.0)
cN1	49 (14.8)	24 (14.7)	25 (15.0)
**Histological type**			
DCIS	36 (10.9)	21 (12.9)	15 (9.0)
IDC	281 (85.2)	133 (81.6)	148 (88.6)
Other	13 (3.9)	9 (5.5)	4 (2.4)
**Tumour subtype**			
HR+/HER2–	215 (65.1)	109 (66.8)	106 (64.3)
Triple-negative	15 (4.5)	4 (2.4)	11 (6.5)
HER2+	64 (19.3)	29 (17.7)	35 (20.9)
Not available	36 (10.9)	21 (12.8)	15 (8.9)
**Surgical procedure**			
SLNB	233 (70.6)	119 (73.0)	114 (69.3)
SLNB + ALND	97 (29.4)	44 (27.0)	53 (31.7)
**NACT**			
Yes	79 (23.9)	36 (22.1)	43 (25.7)
No	251 (76.1)	127 (77.9)	124 (74.3)
**NACT regimen**			
Anthracycline plus taxane	40 (50.6)	19 (52.8)	21 (48.8)
Taxane-based	39 (49.4)	17 (47.2)	22 (51.2)
Not applicable	251	127	124

Values are *n* (%) unless indicated otherwise. UC group, ultrasound-assisted carbon nanoparticle suspension (CNS) sentinel lymph node (SLN) mapping; GC group, dual tracer-guided SLN mapping using CNS plus indocyanine green. Tis, carcinoma in situ; T1, breast tumour 2 cm or smaller; T2, breast tumour larger than 2 cm but at most 5 cm; T3, breast tumour larger than 5 cm; DCIS, ductal carcinoma in situ; IDC, invasive ductal carcinoma; HR, hormone receptor; HER2, human epidermal growth factor receptor 2; SLNB, SLN biopsy; ALND, axillary lymph node dissection; NACT, neoadjuvant chemotherapy.

In the primary endpoint analysis, the SLN identification rate was 94.5 (95 per cent c.i. 90.9 to 98.0) per cent in the UC group and 95.8 (92.7 to 98.9) per cent in the GC group. There thus was a difference of –1.3 (–5.9 to 3.3) per cent, which was lower than the prespecified non-inferiority margin of 6 per cent (*P*_non–inferiority_ = 0.024). Eighty-six (26.1 per cent) of the 330 participants had at least one metastatic SLN. No significant difference was observed in the metastasis rate (30.5 *versus* 24.4 per cent; *P* = 0.222), median number of SLNs harvested (3 (range 1–7) *versus* 3 (1–8); *P* = 0.181), or duration of surgery (mean(s.d.) 7.53(2.77) *versus* 7.63(3.27) min; *P* = 0.316) between the groups (*Table 2*).

**Table 2:** Identification rate and diagnostic performance

	UC group (*n* = 163)	GC group (*n* = 167)	P*
**SLN identification rate**	154 of 163 (94.5; 90.9, 98.0)	160 of 167 (95.8; 92.7, 98.9)	0.024
**No. of SLNs, median (range)**	3 (1–7)	3 (1–8)	0.181†
**Metastasis rate**	47 of 154 (30.5; 23.2, 37.9)	39 of 160 (24.4; 17.1, 31.1)	0.222
**Duration of surgery (min), mean(s.d.)**	7.53 (2.77)	7.63 (3.27)	0.316†

Values are *n* (%; 95% c.i.), unless otherwise indicated. UC group, ultrasound-assisted carbon nanoparticle suspension (CNS) sentinel lymph node (SLN) mapping; GC group, dual tracer-guided SLN mapping using CNS plus indocyanine green. *χ^2^ or Fisher’s exact test, except †*t* test.

In the NACT subgroup, the SLN identification rate was 91.7 (82.2 to 100) per cent in the UC group and 90.7 (81.7 to 99.7) per cent in the GC group (*P* = 1.000). Regarding the initial lymph node status, 24 of 36 patients in the UC group and 25 of 43 in the GC group had cN1 disease. There was no significant difference in the rate metastatic SLNs (42.4 *versus* 38.5 per cent; *P* = 0.733), median number of SLNs collected (3 (3–6) *versus* 3 (3–7); *P* = 0.814), or duration of surgery (6.94(2.03) *versus* 7.87(4.12) min; *P* = 0.241) between the two groups (*Table 3*).

**Table 3:** Identification rate and diagnostic performance in neoadjuvant chemotherapy subgroup

	UC group (*n* = 36)	GC group (*n* = 43)	*P*†
**SLN identification rate**	33 of 36 (91.7; 82.2, 100)	39 of 43 (90.7; 81.7, 99.7)	1.000
**No. of SLNs, median (range)**	3 (3–6)	3 (3–7)	0.814‡
**Metastasis rate**	14 of 33 (42.4; 24.6, 60.2)	15 of 39 (38.5; 22.5, 54.4)	0.733
**Duration of surgery (min), mean(s.d.)**	6.94 (2.03)	7.87 (4.12)	0.241‡
Initial lymph node status*			0.437
cN0	12 of 36 (33.3)	18 of 43 (41.9)	
cN1	24 of 36 (66.7)	25 of 43 (58.1)	

Values are *n* (%; 95% c.i.), unless otherwise indicated; *values are *n* (%). UC group, ultrasound-assisted carbon nanoparticle suspension (CNS) sentinel lymph node (SLN) mapping; CG group, dual tracer-guided SLN mapping using CNS plus indocyanine green. †χ^2^ or Fisher’s exact test, except ‡*t* test.

There were no instances of tracer-related allergic reactions, local inflammatory reactions, or skin or fat necrosis during or after the operation.

## Discussion

This open-label, non-inferiority, phase III RCT demonstrated that the SLN identification rate of intraoperative ultrasound-assisted CNS mapping was non-inferior to that of dual tracer-guided SLN mapping with CNS plus ICG (94.5 *versus* 95.8 per cent), with a comparable number of SLNs harvested and a similar duration of surgery. Furthermore, the SLN identification rate after ultrasound-assisted CNS mapping was also equivalent to that of dual tracer-guided mapping in the NACT subgroup.

Previous anatomical studies have revealed that SLNs are neither evenly nor randomly distributed in the axilla but are located in predetermined anatomical regions. Clough *et al*.^18^ found that the SLNs in 86.8 per cent of patients with breast cancer were located in the area adjacent to the LTV, extending from the lower border of the axilla to the second intercostobrachial nerve, and, more noticeably, 98.2 per cent of SLNs were found in the medial part of the axilla, alongside the LTV, regardless of the site of the primary tumour. These results are consistent with those of a previous autopsy study^19^ in which 87 per cent of axillary SLNs were located between the lateral border of the pectoralis major muscle and the thoracoepigastric vein. Considering the close proximity of the LTV to the lateral border of the pectoralis major muscle, the present study used the latter as a convenient surface landmark for the localization of axillary SLNs^19–21^.

Intraoperative ultrasound-assisted CNS mapping had an SLN identification rate comparable to that of dual tracer-guided mapping with CNS plus ICG. Currently, the standard method for detecting SLNs is a dual tracer-guided technique using technetium-labelled nanocolloid and blue dye^10,22^. A meta-analysis^1^ of 8059 patients from 69 trials documented an SLN identification rate of 91.9 per cent and a false-negative ratio of 7.0 per cent for mapping using radioisotope plus blue dye. Among patients from the AMAROS, ALMANAC, and NSABP-32 trials, the SLN identification rate of the dual technique ranged between 97 and 98 per cent^2,23,24^.

Use of radioisotope in SLN mapping is not without limitations, and so alternative methods for identification have emerged. Contrast-enhanced ultrasound imaging (CEUS) has shown an SLN identification rate of 87.7–89.0 per cent. CEUS also offers unique potential for preoperative identification of metastatic involvement of SLNs via ultrasound imaging^25,26^. Superparamagnetic iron oxide is a safe and non-toxic replacement for radioisotope, with an SLN rate of 94.4–97.4 per cent^12,27^. There is extensive research on ICG as a safe and inexpensive SLN mapping tracer that enables vivid visualization of lymphatic tracts. A meta-analysis^12^ of 21 RCTs suggested that SLNs identification rates are comparable for ICG and radioisotope when used in conjunction with blue dye. According to a recently published prospective observational study^28^, ICG has performance parameters comparable to those of the standard using radioisotope.

The duration of surgery in the GC group was similar to that in the UC group. Although the dye-guided method enables direct visualization of SLNs by distinct colour recognition, visibility can be easily compromised by dense fat layers, rapid transition, or intraoperative bleeding. Moreover, signals from dye-only tracers cannot penetrate the dermis and so the location of SLNs cannot be determined before skin incision. Surgeons sometimes have to increase the length of the incision or spend more time identifying black-stained lymph nodes when using dye alone. Using an NIR camera to detect the fluorescence of ICG, researchers have achieved an identification rate of lymphatic vessels of 57 and 100 per cent in different studies^12^. Based on lymphatic drainage, surgeons can accurately and easily identify SLNs, reduce the duration of surgery, and avoid overtreatment. Given that ICG penetrates tissues to a depth of no more than 2 cm, it should be used with caution in patients with thick tissue layers^29^. In addition, the ICG technique requires a specific NIR camera. The development of high-frequency ultrasound imaging has allowed portable probes to be used increasingly in ultrasound-guided surgery. In the present study, ultrasonography was applied as a non-invasive technique to assist in the intraoperative localization of SLNs. Generally, SLNs can be found in the typical anatomical location in the axilla. With ultrasound assistance, the site of incision and depth of dissection can be determined before operation, and real-time visualization during surgery facilitates the SLNB procedure. Surgeons can master the technique with simple training, and identify the black-stained SLNs swiftly and accurately.

The present study also investigated the identification rate of SLNB after NACT. In the NACT subgroup, the identification rate was comparable in the UC and GC groups (91.7 *versus* 90.7 per cent), in line with previous studies^6–9^. An increased identification rate and decreased false-negative rate in dual tracer-guided SLNB has been demonstrated, when more than three SLNs including the clip-marked lymph nodes are removed^6–9^. In the subgroup analysis of patients who had undergone NACT in the present study, the identification rates among patients with cN0 disease at baseline were 100 per cent in both groups, and patients in whom the SLN was not identified were concentrated to those with cN1 disease at baseline.

There are some limitations to this study. Dual tracer-guided techniques comprising radioisotope and blue dye remain the current standard for detecting SLNs. Owing to the strict limitations of application of radioisotope, the control group in this study underwent dual tracer-guided mapping with CNS plus ICG. Moreover, not all patients had ALND for ethical reasons, so data on the false-negative rate could not be acquired. All patients were enrolled from the same hospital, although randomized to four independent surgical teams. Further multicentre studies are required to validate the results of this study. In addition, the Chinese population has low BMI, and the findings of this study should be applied with caution to populations with higher BMI. Finally, the application of ultrasound-assisted CNS mapping in patients who have received NACT requires further experimental verification as the present data are from a subgroup analysis.

The diagnostic performance of ultrasound-assisted CNS mapping was non-inferior to that of dual tracer-guided SLN mapping with CNS plus ICG in patients with early breast cancer. Compared with single-tracer mapping, the ultrasound-assisted technique facilitated SLNB and has potential clinical value in patients treated with NACT.

## Supplementary Material

znac311_Supplementary_Data
