# Supplementary material for: Ultrasound-assisted carbon nanoparticle suspension mapping versus dual tracer-guided sentinel lymph node biopsy in patients with early breast cancer (ultraCars): phase III randomized clinical trial
Source: Br J Surg. 2022 Sep 8;109(12):1232–8. doi: 10.1093/bjs/znac311 (PMC10364740; doi:10.1093/bjs/znac311)
Supplement: znac311_Supplementary_Data [file znac311_supplementary_data.zip › Protocol.docx]

**TITLE:** **Ultrasound-assisted carbon nanoparticle suspension mapping versus dual-tracer-guided sentinel lymph node biopsy in patients with early breast cancer (UltraCars):**

**A prospective, randomized, controlled, phase 3 trial**

**Investigator Initiated Clinical Study Protocol No. 20190511**

| **Coordinating Center:** | Guangdong Provincial People’s Hospital, Guangdong Academy of Medical Sciences |
| --- | --- |
| **Principal Investigator:** | Kun Wang  Telephone number: 00-86-83827812-80420  E-mail: [wangkun@gdph.org.cn](mailto:wangkun@gdph.org.cn) |
| **Co-Investigators:** | Liulu Zhang,  Telephone number: 00-86-83827812-80421  E-mail: [zhangliulu@gdph.org.cn](mailto:zhangliulu@gdph.org.cn) |
| **Biostatistician:** | Qi Ge  Central Research Institute, United Imaging Health care Group Co., Ltd, Shanghai, China  E-mail: [Qige@126.com](mailto:Qige@126.com) |
| **Study Coordinator:** | Minyi Cheng  Telephone number:00-86-83827812-50760  E-mail: [chengminyi@gdph.org.cn](mailto:chengminyi@gdph.org.cn) |

**Protocol Type/Version #/Version Date:** Original/Version 1.1/May 11, 2019

**SYNOPSIS**

| **Study Title** | Ultrasound-assisted carbon nanoparticle suspension mapping versus dual-tracer-guided sentinel lymph node biopsy in patients with early breast cancer (UltraCars): a prospective, randomized, controlled, phase 3 trial |
| --- | --- |
| **Clinical Phase** | Phase III |
| **Centers in which trial will be carried out** | Guangdong Provincial People's Hospital, Guangdong Academy of Medical Sciences (Guangzhou, China). |
| **Clinical Research Ethics Committees which have approved the study** | This study was approved by the Research Ethics Committee (No. GDREC2019610H) |
| **Good Clinical Practice (GCP) Study Monitors** | Jinfeng Yan: gdphgcp@gdph.org.cn  Pianpian Liu: pianpian.liu@medpison.cn  Danni Zhou: danni.zhou@linkstart.com.cn  Lanmei Wu: lanmei.wu@medpison.cn |
| **Objectives** | **Principal objective:**  To compare the identification rate of sentinel lymph nodes (SLNs) using an ultrasound-assisted carbon nanoparticle suspension mapping versus dual-tracer-guided sentinel lymph node biopsy in patients with early breast cancer, defined as the number of patients whose SLNs were detected divided by the total number of patients included.  **Secondary objectives:**   - Median number of SLNs collected; - The metastatic rate of SLNs, defined as the number of patients who have at least one positive SLN divided by the number of patients whose SLNs were identified; - Operative duration, defined as the time from skin incision to resection of the SLN specimens; - Intraoperative or postoperative complications.   **Exploratory objectives:**  The identification rate of SLNs in patients after neoadjuvant therapy. |
| **Study design** | This study is a single-centre, open-label, randomized controlled, non-inferiority, phase 3 trial. Patients will be randomly assigned (1:1) using a permuted block randomization scheme to receive either ultrasound-assisted CNS (UC group) or CNS plus ICG dual-tracer-guided (GC group) SLN mapping.   - UC group: Intraoperative ultrasound-assisted CNS will be used for SLN mapping. - GC group: CNS plus ICG dual-tracer-guided SLN mapping.   All removed lymph nodes will be sent for intraoperative frozen section analysis. Axillary lymph node dissection will be undertaken according to the pathologic findings of the SLNs and the current international guidelines. |
| **Inclusion criteria** | 1. The patient must be female and 18 years of age or older.  2. Resectable invasive adenocarcinoma of the breast, histologically confirmed by core or open biopsy.  3. Ductal carcinoma in situ histologically confirmed by core or open biopsy and scheduled for mastectomy.  4. The breast cancer must be preoperative clinical Tis, T1, T2, T3, M0.  5. Without clinical or radiological nodal involvement (cN0): no positive ipsilateral axillary lymph nodes; no prior removal of ipsilateral axillary lymph nodes; no suspicious palpable nodes in the contralateral axilla or palpable supraclavicular or infraclavicular nodes, unless proven nonmalignant by biopsy.  6. With clinical positive lymph nodes (cN1) (including any abnormal or enlarged clinically palpable lymph nodes or core biopsy/surgical biopsy/FNA evidence of malignant cell within any lymph nodes) that was downstaged to cN0 following neoadjuvant therapy.  7. The patient must have an ECOG performance status of Grade 0-1.  8. The patient must provide written informed consent before participating in the study. |
| **Exclusion criteria** | 1. The breast has ulceration, erythema, infiltration of the skin or underlying chest wall (complete fixation), peau d'orange, or skin edema of any magnitude. Tethering or dimpling of the skin and nipple inversion is allowed.  2. The patient has a known hypersensitivity to tracers planned for use during SLNB.  3. Other prior breast malignancy except for lobular carcinoma in situ.  4. The patient has had prior breast implants.  5. The patient has had prior breast reduction surgery.  6. The patient has had other prior surgery in the upper, outer quadrant, areola, or axilla on the side of the affected breast.  7. The patient has a positive pregnancy test or is lactating.  8. The patient has participated in another investigational drug study during the 30 days prior to signing informed consent. |
| **Study population and total number of patients** | PASS 2019 software was used to calculate the required sample size. The non-inferiority (one-sided) hypothesis is adopted, which assumed an SLN identification rate of 96% for each group, and a non-inferiority margin (∆) of 6% to secure an identification rate above 90% (one-sided test significance level (α) =0.05). A sample size of 264 patients was required to give a power of 80% to reject the null hypothesis that the identification rate in the UC group was inferior to that of the GC group by more than a 6% non-inferiority margin (with a 5% probability of type I error). Anticipating a 10% dropout rate, at least 294 patients will need to be recruited. |
| **Timing and planned completion date** | **Initiation of inclusion:** December 2019  **Completion of inclusion:** May 2021  **Final analysis:** Third quarter 2021 |

**STUDY DESIGN**

This study is a single-centre, open-label, randomized controlled, non-inferiority, phase 3 trial. The patients will be randomly assigned (1:1) using a permuted block randomization scheme to receive either ultrasound-assisted CNS (UC group) or CNS plus ICG dual-tracer-guided (GC group) SLN mapping.

UC group=ultrasound-assisted CNS for SLN mapping. GC group=dual-tracer method using CNS plus ICG for SLN mapping. DCIS=ductal carcinoma in situ. ALND=axillary lymph node dissection. CNS=carbon nanoparticle suspension. ICG=indocyanine green.

**PROTOCOL SIGNATURE PAGE**

**Ultrasound-assisted carbon nanoparticle suspension mapping versus dual-tracer-guided sentinel lymph node biopsy in patients with early breast cancer (UltraCars):**

**A prospective, randomized, controlled, phase 3 trial**

I have reviewed and approved this protocol. My signature assures that this study will be conducted according to all stipulations of the protocol, including all statements regarding confidentiality.

Approved by:

President of the UltraCars Trial

**Prof. Dr. Kun Wang**

**Signature: _____________________________________ Date: ___________**

Approved by:

Co-Investigators

**Dr. Liulu Zhang**

**Signature: _____________________________________ Date: ___________**

Approved by:

Good Clinical Practice (GCP) Study Monitors

**Dr. Jingfeng Yan**

**Signature: _____________________________________ Date: ___________**

**1. INTRODUCTION**

**1.1 Background**

**1.1.1 Lymphatic Mapping and Sentinel Lymph Node Biopsy**

The current treatment of breast cancer involves various combinations of multiple treatment modalities including surgery, radiation and systemic therapy. Traditionally, regional lymph node status was thought to be the most important determinant of the required extent of surgery and the need for systemic and radiation therapy^1^. Axillary lymph node dissection (ALND) is, however, associated with significant morbidity (lymphedema, pain and decreased range of motion postoperatively). Therefore, sentinel lymph node biopsy (SLNB) has become the standard of care in clinically node-negative (cN0) early breast cancer (EBC) due to its low false-negative rates (FNRs), without impairing local control or survival^2,3^. This less extensive staging procedure has allowed safe omission of ALND in approximately 60% of patients with no pathological evidence of disease in the excised sentinel lymph nodes (SLNs)^4^.

Since their first descriptions during breast cancer by Krag in 1993^5^ and Giuliano in 1994^6^, intraoperative lymphatic mapping and SLNB have become standard-of-care technologies widely utilized in the surgical staging and treatment of breast cancer. The utilization of SLNB for breast cancer has resulted in a reduction in the short-term complications and long-term morbidities, including lymphedema, associated with ALND^7-10^. Additionally, the utilization of SLNB has allowed for the avoidance of ALND in cases of early-stage breast cancer with limited SLN involvement in both cases of breast-conserving surgery and mastectomy^11,12^.

An ideal lymph node mapping agent for SLNB should exhibit rapid clearance from the injection site, rapid uptake and high retention within the first draining lymph nodes, and low uptake by subsequent, higher echelon lymph nodes. The ideal lymph node mapping agent should also have biochemical purity, molecular size uniformity, and a high level of biological and radiation safety. Specifically, a radiopharmaceutical lymph node mapping agent should possess characteristics allowing for convenient, rapid, and stable ^99m^Tc labeling.

At present, gold standard lymphatic mapping uses a combination of blue dye (BD; patent blue, methylene blue, or isosulfan blue) and radioactive colloid^13-16^. This dual tracer approach facilitates high sensitivity and low FNR^14^. However, BD can cause allergic reactions in 1.8% of patients, among which approximately 23% are type I hypersensitivity and 69% are type IV skin reactions^17^. Additionally, BD can cause semipermanent skin staining/tattooing, which may or may not fade after several months^18^, and the technique may fail to accurately identify all sentinel nodes when used in isolation^19^. Radioactive colloids expose both the patient and staff to radiation^20,21^, require a constant supply due to their short shelf-life (but there are limited numbers of nuclear reactors capable of making medical-grade isotopes)^22^, may not be widely available to all hospitals, mandate special licensing and hospital infrastructure for safe use and disposal as per the Ionizing Radiation Medical Exposure Regulations^23^, and, when used in isolation, fail to give a visual cue for nodal stations. The limitations of both dye and radioisotope (RI) mapping have led to the development of new contrast agents for SLNB, such as magnetic^24,25^ and fluorescence imaging approaches^26-36^.

In the era of minimally invasive surgery for breast cancer, the good benefits of neoadjuvant chemotherapy (NCT) have encouraged surgeons to adopt more conservative surgical approaches to reduce the incidence of complications and adverse reactions further. After NCT, approximately 21.1% (10.1-74.2%) of patients are able to achieve a pathological complete response (pCR)^37^, among which the axillary pCR rate is between 23-74%^38,39^. The FNR of SLNB after NCT in breast cancer has been reported to be 8-14.2%^37^. Currently, the American Society of Breast Surgeons recommends that SLNB FNR be controlled at approximately 5%^40^, which suggests that the method should be improved to reduce the FNR. The current NCCN guidelines suggest that the goals of neoadjuvant therapy include reducing the possibility of axillary breast cancer in the retained axillary lymph nodes, but the precise resection of metastatic axillary lymph nodes is an important issue in axillary surgery. For axillary metastatic lymph nodes confirmed by pathological biopsy before neoadjuvant therapy, a clip can be inserted to locate them after NCT, which allows them to be completely removed by SLNB, which is the primary concept of targeted axillary resection (TAD)^41^. The results of the ACOSOG Z1071 clinical trial showed that cases with clip-labeled biopsy greater than or equal to 3 SLNs (double tracer) were associated with a lower FNR (clip-labeled FNR: 6.8%; unlabeled FNR: 13%)^42^. Before NCT, the lymph nodes were labeled with radioactive iodine 125 particles. After NCT, targeted surgery was performed to control the FNR at 7%^43^. TAD with radioactive iodine 125 particles was performed at the MD Anderson Center to further reduce the FNR to 2%^44^. In a clinical trial evaluating the feasibility of intraoperative ultrasound-guided removal of clip-labeled SLNs, 44 (96%) of 46 cases were successful in the removal of clip-labeled axillary metastatic lymph nodes. The overall FNR was 4.1% (95% CI 0.1-21.1%)^45^.

**1.1.2 Alternative Methods for SLN Mapping**

Contrast-enhanced ultrasound (CEUS) using microbubbles is a new technique that potentially allows SLNB to be performed outside the operating theatre. Three studies followed the same protocol and showed an SLN identification rate between 87.7% and 89%^46-48^. The most recent and largest cohort study^48^ involving 347 patients was the only study that attempted to accurately calculate the sensitivity of the technique, for which they identified 22 false-negative results, providing a sensitivity of only 61%. Further refinement of this system with improved sensitivity could provide an alternative to surgical sentinel lymph node biopsy.

Superparamagnetic iron oxide (SPIO) has been used as an MRI contrast agent for preoperative assessment of sentinel lymph nodes with better results than other imaging modalities such as PET^49,50^. The study by Shiozawa and colleagues^51^ considered the performance of SLNB in breast cancer patients using SPIO and a handheld magnetometer. This study was limited by its small size and the comparison of SPIO to blue dye only as opposed to a dual technique but it showed SPIO to be more sensitive than blue dye, albeit significantly below the accepted sensitivity for the dual technique. However, even in this study, the FNR of the magnetic technique (8%) was twice that of the standard technique (4%) based on the 25 patients who were node-positive after sentinel lymph node biopsy (no compulsory axillary node clearance). Additionally, the authors identified a discordance rate of 6.9% for nodes identified by the two techniques. This finding suggests that the magnetic technique does not always identify the same nodes as the dual technique, which might be a limitation because it leads to false-negative staging. However, this situation could represent differences in the standard techniques used by the centers within this trial.

The potential benefits of SPIOs include their half-life of several years, which enables them to be shipped to remote locations worldwide. They do not need special handling procedures, which eliminates the issues of scheduling with a nuclear medicine department and safe waste disposal. SPIOs are not believed to be toxic or dangerous in clinical use. SPIO can be injected intravenously or interstitially, and they are taken up by macrophages in the mononuclear phagocyte system of the liver, spleen, lymphatic system and bone marrow and broken down to be distributed across iron stores in the body^52^. Their main drawback is the need to remove metal retractors from the wound when using the magnetometer to take readings during surgery. Further assessment of this technique is warranted in larger studies against the dual technique.

There is a growing body of literature supporting the role of indocyanine green (ICG) dye in lymphatic mapping. ICG is a fluorescent dye that can be detected using near infrared cameras. The use of ICG in conjunction with a near infrared camera offers many potential advantages over the use of other techniques, including the ability to inject the material while the patient is under anesthesia in the operating room, the ability to directly visualize lymphatic flow using near infrared imaging, and no special requirement for material handlings. ICG is approved by FDA for vascular fluorescence and liver function studies in humans. ICG is a heptamethine indocyanine with moderate optical properties which that emits 800 nm. There are many uses for ICG during image-guided surgery. After intravenous injection, it can be used for near-infrared (NIR) angiography of blood vessels, identification of the extrahepatic bile ducts, and identification of liver metastases. After subcutaneous injection, ICG has been reported by numerous authors to be safely used for SLN mapping^53-57^ and assessing lymphatic function^58^. ICG has been used for the last 50 years as a visible dye (green for ICG), and there is an unprecedented body of clinical data regarding its safety when used at millimolar concentrations^58^. The use of ICG for lymphatic mapping in breast cancer patients has been demonstrated to be safe in numerous human studies^59-63^. A study from Europe demonstrated high concordance between the number of nodes identified with ICG and ^99m^Tc-labeled radiotracers^63^.

**1.1.3 Carbon Nanoparticle Suspensions (CNS)**

Another promising sentinel lymph node tracer is carbon nanoparticle suspensions (CNS). With advancements in nanotechnology, CNS could be widely used in surgeries. CNS, which contains particles with a diameter of 150 nm, can easily pass through the lymphatic vessels (diameter, 120–500 nm) but are unable to pass through the blood capillaries (diameter, 20–50 nm). Previous studies have demonstrated the safety of CNS^64^. In recent years, CNS has been used as a lymph node tracer during surgeries for thyroid cancer, gastric cancer, and colorectal cancer^65-67^.

Our previous study showed that CNS is a nonallergic sentinel lymph node tracer that has a good detection rate (99%)^68^. CNS has a large particle diameter, so it has difficulty entering blood vessels. In addition, CNS can readily pass through the lymphatic vessels and ultimately accumulate in the lymph nodes, staining them black for easy recognition. The CNS dye stays in the lymph nodes for an extended time, giving the surgeon a sufficient window for SLN detection. Moreover, CNS does not cause black staining of the surgical area. However, surgeons do need a certain amount of time to practice and master this staining method for sentinel lymph node biopsy.

**1.1.4 Ultrasound-guided Surgery**

ICG and RI are able to provide deep insight into the SLNB location prior to skin incision. However, when the CNS technique is used alone for SLNB, it depends on the surgeon's naked-eye observation and experience, which may prolong the duration of surgery. Therefore, locating black-stained lymph nodes during surgery still requires improvement.

An easily available and feasible method to improve the amount of healthy breast tissue spared while ensuring tumor-free resection margins is intraoperative ultrasonography (US). US-guided surgery enables the surgeon to visualize the tumor during excision. Previous studies have clearly shown the efficacy of US-guided surgery for nonpalpable tumors. Rahusen et al. reported that US-guided surgery, during which an experienced radiologist performed the US, is superior to wire-guided surgery with respect to tumor-free resection margins (89% and 55% of cases, respectively). Snider et al. also showed an excellent rate of tumor-free resection margins using USS (82%) with a smaller volume of healthy breast tissue resection compared to wire-guided surgery (62.2 cm^3^ and 81.1 cm^3^, respectively)^69-74^.

Previous anatomical studies have revealed that the SLNs are neither evenly nor randomly distributed in the axilla but are instead located in predetermined anatomical regions. K.B. Clough et al. found that the SLNs in 86.8% of breast cancer patients were located in the area adjacent to the lateral thoracic tributary of the axillary vein (LTV), extending from the lower border of the axilla to the second intercostobrachial nerve (ICBN), and, more noticeably, 98.2% of SLNs were found in the medial part of the axilla, alongside the LTV, regardless of the site of the primary tumors^75^. These results are consistent with a previous autopsy study in which 87% of axillary SLNs were located between the lateral border of the pectoralis major muscle and the thoracoepigastric vein^76^. Considering the close proximity between the LTV and the lateral border of the pectoralis major muscle^76-68^, the present study used the lateral border of the pectoralis major muscle as a convenient surface landmark for the localization of axillary SLNs.

Ultrasound is a noninvasive method that can not only be used to check whether axillary lymph nodes have metastasized before surgery but can also help to determine the anatomical locations of suspicious lymph nodes. Based on these findings, the following questions have arisen: Can the CNS be used as a tracer for sentinel lymph node biopsy with the help of ultrasound to identify sentinel lymph nodes in the conventional anatomical position? Can ultrasound replace ICG or RI for locating sentinel lymph nodes?

**1.2 Rationale**

The purpose of this prospective, randomized controlled phase 3 trial is to compare the feasibility and diagnostic performance of ultrasound-assisted carbon nanoparticle suspension mapping versus dual-tracer-guided sentinel lymph node biopsy in patients with early breast cancer.

**2. STUDY OBJECTIVES/ENDPOINTS**

**2.1 Principal Objective**

- To compare the identification rate of SLNs by ultrasound-assisted carbon nanoparticle suspension mapping versus dual-tracer-guided sentinel lymph node biopsy in patients with early breast cancer.

**2.2 Secondary Objectives**

- To compare the median number of SLNs collected;
- To compare the metastatic rate of SLNs, defined as the number of patients who have at least one positive SLN divided by the number of patients whose SLNs were identified;
- To compare the operative duration, defined as the time from skin incision to resection of the SLN specimens in patients with at least one SLN detected;
- To compare the intraoperative or postoperative complications.

**2.3 Exploratory Objectives**

- To compare the identification rate of SLNs in patients after neoadjuvant therapy.

**3. PATIENT SELECTION**

Up to 294 evaluable subjects (147 per cohort) will be enrolled in this trial. Evaluable subjects will be randomly assigned (1:1) using a permuted block randomization scheme to receive either ultrasound-assisted CNS (UC group) or CNS plus ICG dual-tracer-guided (GC group) SLN mapping. Determination of the study eligibility will be established by the investigators on the basis of the inclusion and exclusion criteria.

The inclusion/exclusion criteria were designed to ensure that the study subjects have a diagnosis of primary breast cancer and were candidates for surgical intervention as a treatment for breast cancer.

Subjects who meet all of the inclusion criteria and none of the exclusion criteria will be eligible for enrollment in this trial. Written, dated informed consent will be obtained from all subjects before enrollment in the study.

**3.1 Inclusion Criteria**

Patients meeting all of the following inclusion criteria by the end of the screening phase should be considered for admission to the study:

1. The patient must be female and 18 years of age or older.

2. Resectable invasive adenocarcinoma of the breast, confirmed histologically.

3. Ductal carcinoma in situ confirmed by histologically scheduled mastectomy.

4. The patient must have preoperative clinical Tis and T1, T2, T3, M0 breast cancer.

5. Without clinical or radiological nodal involvement (cN0): no positive ipsilateral axillary lymph nodes; no prior removal of ipsilateral axillary lymph nodes; no suspicious palpable nodes in the contralateral axilla or palpable supraclavicular or infraclavicular nodes, unless proven nonmalignant by biopsy.

6. Clinically positive lymph nodes (cN1) (including any abnormal or enlarged clinically palpable lymph nodes or core biopsy/surgical biopsy/FNA evidence of malignant cells within any lymph nodes) were downstaged to cN0 following neoadjuvant therapy.

7. The patient must have an ECOG performance status of Grade 0-1.

8. The patient must provide written informed consent before participating in the study.

**3.2 Exclusion Criteria**

Patients will not be entered into the study if they meet any of the following exclusion criteria:

1. The breast has ulceration, erythema, infiltration of the skin or underlying chest wall (complete fixation), peau d'orange, or skin edema of any magnitude. Tethering or dimpling of the skin or nipple inversion is allowed.

2. The patient has known hypersensitivity to any of the tracers planned for use during SLNB.

3. Other prior breast malignancies except for lobular carcinoma in situ.

4. The patient has had prior breast implants.

5. The patient had prior breast reduction surgery.

6. The patient had other prior surgery in the upper, outer quadrant, areola, or axilla on the same side as the affected breast.

7. The patient had a positive pregnancy test or was lactating.

8. The patient participated in another investigational drug study during the 30 days prior to signing informed consent.

**4. RANDOMIZATION TO THE TREATMENT ARM**

The participating investigators will be responsible for ensuring that each new patient fulfills the study inclusion criteria, is informed appropriately about the study, reads and understands the patient information sheet and dates and signs the informed consent form for participation in the study.

An interactive response system will be used to achieve treatment assignment. A randomization sequence will be created with a 1:1 allocation. Randomization envelopes, numbered with sequential subject IDs, will be prepared by the research office personnel. Each envelope will contain a card identifying the test type assigned to that subject ID. The randomization envelopes will be maintained in a secure fashion in the research office.

**4.1 Timing of Registration and Randomization to Study**

Eligible patients will sign the informed consent form to agree to participate in the study. The randomization should be completed at least one working day in advance of the planned operation date. No more than 14 days must elapse between the date of treatment randomization and the date of operation. The interval between Day 1 of the last cycle of neoadjuvant chemotherapy and randomization to the study could be no more than 8 weeks (2 months).

The study monitor will notify the investigator by electronic mail, within a period of one working day, of the patient study number and treatment arm to which she has been randomized. This study is unmasked, and all patients, investigators and surgeons will be aware of the study group assignment.

The administrative coordinator of the study registered all selected patients before beginning the operation. Patients who were not registered before the operation will not be permitted to participate in the study.

**4.2 Treatment Groups**

Patients will be assigned by the randomization system to one of the following groups:

- UC group: ultrasound-assisted CNS.
- GC group: CNS plus ICG dual-tracer-guided.

**5. PATIENT TREATMENT**

**5.1 Preoperation Chemotherapy**

Standard neoadjuvant therapy before randomization will be performed according to the NCCN guidelines and clinical practice.

Evaluation of neoadjuvant chemotherapy (every 2 cycles):

- Breast and axillary ultrasound (every 2 cycles)
- Mammogram (first plus one before surgery)
- Breast MRI (first plus one before surgery)

Quality control of SLNB after neoadjuvant therapy entails removal of the clip-containing node and at least 3 lymph nodes. Intraoperative radiography will be used to identify the clipped node.

**5.2 Procedures**

After completing preoperative radiographic evaluation, participants/patients will proceed to surgery for SLNs mapping. Excision of the primary tumor will be performed at the same time and in the same setting.

**Experimental arm (****UC group):**

One milliliter of CNSs will be subcutaneously injected into the areolar area in the upper outer quadrant of the breast. The injection site will be massaged for 15 minutes to promote drainage of the tracer into the axilla. During the intraoperative ultrasound-assisted procedure, an ultrasound diagnostic system will be used. Before making the incision, ultrasound-guided exploration of the SLNs will be performed by placing the probe on the lateral border of the breast and sliding it cranially along the lateral border of the pectoralis major muscle. A sterile skin marker will be used to mark the optimal site of incision over the targeted lymph nodes, and the distance from the skin to the nodes will be measured by ultrasound and recorded in millimeters. Blunt dissection is carried out to identify the CNS-stained nodes around the marked region. An ultrasonography probe is placed repeatedly in or around the wound at different angles for adequate visualization if the SLNs could not be localized with further dissection. All black-stained lymph nodes or suspicious lymph nodes will be excised.

**Control arm (GC group):**

One milliliter of CNSs will be subcutaneously injected into the areolar area in the upper outer quadrant of the breast. The injection site will be massaged for 15 minutes to promote drainage of the tracer to the axilla. One milliliter of diluted ICG (2.5 mg/ml) will be subsequently injected into the areolar area in the upper outer quadrant of the breast. A fluorescence tracer system will be used to visualize the subcutaneous lymph vessels and localize the SLNs. All fluorescent or black-stained lymph nodes along with any suspicious nodes will be removed. The remaining surgical field will be reexamined to ensure complete resection of the fluorescent lymph nodes.

An SLN number will be collected immediately following excision of each lymph node:

- A sentinel lymph node is defined as any lymph node that is either “fluorescent” and “black”, “fluorescent” only, “black” only, or a palpable suspicious lymph node.
- A “fluorescent” SLN is defined as a node that emits fluorescence under near-infrared camera detection.
- A “black” SLN is defined as any lymph node that is visibly stained black, has a visible contiguous black-stained afferent lymphatic, or both.
- Digital palpation (for the detection of intraoperatively clinically grossly suspicious (i.e., enlarged or hard) lymph nodes) will also be conducted.

**5.3 Reagents and Equipment**

- The dilution concentration of CNS (Lai Mei Pharmaceutical Co, Chongqing, China) is 50 mg/ml.
- The dilution concentration of ICG (Medical Pharmaceutical Co, Dandong, China) is 2.5 mg/ml.
- A fluorescence tracer system (Dipu Medical Technology Co., Ltd, Zhuhai, China) will be used to track subcutaneous lymph vessels and localized sentinel lymph nodes.
- An ultrasound diagnostic system (TOSHIBA APLIO 400, Japan) will be used for ultrasound positioning to locate lymph nodes.

CNSs and ICG will be obtained from commercial sources and stored according to the package insert instructions. The investigator is responsible for accounting for all trial drugs. Each time CNSs and ICG are dispensed to subjects enrolled in this study, the following information at a minimum will be recorded on the drug accessibility log: the patient’s subject number, manufacturer, and lot or batch number. At the completion or termination of the trial, a final drug accountability review and reconciliation must be completed, any discrepancies must be investigated and their resolution documented.

**5.4 Intra-operative Frozen Section of Lymph Nodes**

All removed lymph nodes will be sent for intraoperative frozen section analysis and pathology for further evaluation. Prior to any pathological evaluation, confirmation of ex vivo counts per tissue should be conducted. The pathological evaluation of the lymph nodes will include serial sectioning with H&E staining as well as IHC stain.

- In those cases in which intraoperative frozen section analysis is performed and all SLNs are found to be negative for malignancy on intraoperative frozen section evaluation, no further removal of axillary lymph nodes will be undertaken, unless otherwise determined as necessary at the discretion of the operating surgeon.
- In those cases in which intraoperative frozen section analysis is performed and the SLNs are determined to be positive for malignancy on intraoperative frozen section evaluation, a formal axillary lymph node dissection (levels I and II) may be performed at the discretion of the operating surgeon, or may be excluded at the discretion of the operating dissection (as based upon the ACOSOG Z0011 criteria).
- Any axillary lymph node that is neither “fluorescent” nor “black” but is determined to be intraoperatively clinically grossly suspicious (i.e., enlarged or hard) will also be removed. In those cases in which any clinically grossly suspicious lymph nodes removed intraoperatively are determined to be positive for malignancy on intraoperative frozen section evaluation, a formal axillary lymph node dissection may be performed at the discretion of the operating surgeon.
- In those cases in which no SLN can be intraoperatively identified at the time of the SLNB procedure, formal axillary lymph node dissection may be performed at the discretion of the operating surgeon.

**5.5 Postoperative Procedures**

All excised lymph nodes should undergo histopathological evaluation for the presence of tumors according to the current institutional standards. Final pathology results need to reflect consistent numbering and labeling from the intraoperative worksheets to the final report. SLNs need to be individually identified in the final pathology report.

Telephone follow-up 30 days post-operation will be conducted for adverse event assessment.

**6. STATISTICS**

**6.1 Randomization Methods**

Subjects who meet all inclusion and exclusion criteria will be enrolled in this study. Enrolled subjects will be randomized to one of two treatment groups: the UC group or the GC group. See Section 4 above for specifics on the randomization procedure.

**6.2 Study Populations**

The primary efficacy analysis will be carried out on the intent-to-treat population (ITT), which is defined as the population who underwent SLNB. The ITT analysis will be carried out for the identification rate, the median number of SLNs collected, the operative duration, and the intraoperative or postoperative complications.

**6.3 Statistical Methods**

The statistical analyses for the efficacy and demographic variables are described below. Unless otherwise stated, all hypothesis tests will be conducted using a two-sided α=0.1 (one-sided α=0.05) level of significance.

**6.3.1 Analysis of Baseline and Demographic Characteristics**

Baseline and demographic characteristics of the safe population will be summarized by treatment group and overall. Continuous variables will be summarized via the mean, median, standard deviation, minimum, maximum, and number of nonmissing observations. Categorical variables will be summarized via counts and percentages. The Chi-squared test or Fisher's exact test for categorical parameters and the t-test for continuous parameters will be applied to compare the two intervention groups. The results will be presented as the number of patients (%) or mean ± standard deviation (SD). P<0.05 is defined as statistical significance.

**6.3.2 Analysis of Primary Variables**

The primary endpoint is the identification rate of the SLNs, defined as the number of patients who had at least one SLN detected divided by the total number of patients included. Quantitative variables will be expressed as percentages. The 95% confidence interval will be calculated.

**6.3.3 Analysis of Secondary Variables**

The chi-squared test or Fisher's exact test for categorical parameters and the t-test for continuous parameters will be applied to compare the two intervention groups. The results will be presented as the mean ± standard deviation (SD). P<0.05 is defined as statistical significance.

**The median number of SLNs collected:**

Summary statistics (mean, median, standard deviation, minimum, and maximum) of the number of SLNs will be displayed for each treatment group.

**The metastatic rate of SLNs:**

The metastatic rate of SLNs, defined as the number of patients who have at least one positive SLN divided by the number of patients whose SLNs were identified.

**Operative duration:**

The operative duration is defined as the time from skin incision to resection of the SLN specimens among patients with at least one SLN detected and summary statistics (mean, standard deviation, minimum, and maximum).

**6.3.4 Safety Analyses**

The occurrence of any drug-related adverse events is not expected. The most likely drug-related adverse events include allergic reactions, local inflammatory reactions, and skin or fat necrosis.

An adverse event is any untoward, undesired, and/or unplanned clinical event in the form of signs, symptoms, disease, or laboratory testing, or physiological observations occurring in a human participating in a clinical study with a study drug, regardless of the causal relationship. This includes the following:

- Any clinically significant worsening of a preexisting condition.
- Any recurrence of a preexisting condition.
- An AE occurring from overdose of a study drug whether accidental or intentional.
- An AE occurring from abuse of a study drug.
- An AE that has been associated with the discontinuation of the use of a study drug.

A serious adverse event (SAE) is any AE occurring at any dose that results in one or more of the following outcomes:

- Death.
- Life-threatening situation.
- Inpatient hospitalization or prolongation of an existing hospitalization.
- Persistent or significant disability or incapacity.

**6.3.5 Determination of Sample Size**

The sample size for the study is based on the primary endpoints. The non-inferiority (one-sided) hypothesis is adopted, which assumed an SLN identification rate of 96% for each group^79^ and a non-inferiority margin (∆) of 6% to secure an identification rate above 90% (one-sided test significance level (α) =0.05). A sample size of 264 patients was required to give a power of 80% to reject the null hypothesis that the identification rate in the UC group is inferior to that of the GC group by more than a 6% non-inferiority margin (with a 5% probability of type I error). Anticipating a 10% dropout rate, at least 294 patients will need to be recruited.

**6.3.6 Handling Missing Values**

In the statistical analysis of the efficacy and safety endpoints of the study, any subjects who have missing values for a given endpoint will not be used in the respective analysis.

**6.3.7 Interim Analyses**

There are no formal interim analyses planned for this study.

**7. ETHICAL CONSIDERATIONS**

**7.1 Declaration of Helsinki**

This study will be carried out in accordance with the Declaration of Helsinki, following the Good Clinical Practice Guidelines of the International Conference on Harmonization (GCP/ICH) and in compliance with any applicable legislation.

**7.2 Informed Consent**

The participating investigators will be responsible for ensuring that each new patient fulfills the study inclusion criteria, is informed appropriately about the study, reads and understands the patient information sheet and dates and signs the informed consent form for participation in the study. A signed and dated copy of the informed consent will be provided to the patient. The patient may withdraw from the study at any time, without affecting her future medical care.

**7.3 Confidentiality**

The investigator will be responsible for maintaining the information required for each patient (e.g. initials and name, address, telephone number, social security number and study identity). This information must be kept confidential for the legally stipulated period, as required by Chinese legislation.

**7.4 Clinical Research Ethics Committee (CREC)**

A duly constituted Ethics Committee will review the final approved protocol and informed consent. The decision of the Ethics Committee regarding the realization of the study will be submitted in writing. The investigator agreed to present to the CREC the necessary reports on the study progress, and to report any serious adverse events, life-threatening events or deaths. The investigator will also inform the CREC of any cases of serious adverse events reported in other clinical studies carried out with the study drug. The investigator must inform the CREC of the termination of the study.

**7.5 Protocol Modifications**

A significant protocol amendment is any modification that may affect the realization of the study, the potential benefits to the patient or that may affect patient safety, including changes in the study objectives, study design, patient population, sample size, study procedures or significant administrative changes. The amendment will be authorized by the investigator and approved by the Ethics Committee and the health authorities before implementation, in accordance with local legislation.

A non-significant protocol modification consists of minor corrections and/or clarifications that do not affect the manner in which the study is performed. These non-significant amendments will be authorized by the investigator and will be documented in a memorandum. Non-significant modifications will be reported to the CREC and to the health authorities, in accordance with local legislation.

**7.6 Data Collection**

UltraCars will provide case report forms (CRFs) in which the investigators can record patient study data. The monitor must verify the CRFs, comparing them with the source data (clinical records, specialist medical reports, etc). Printed copies of all CRFs from patients included in the study in each surgical group will be provided after completion of the study.

**7.7 Patient Identification**

In the initial visit, the initials and date of birth of the patients screened for the study will be recorded chronologically in the investigator’s file. If any patient is excluded from participation in the study, the reason for this must be documented in the space provided. A study number will be assigned to each patient at the time of registration. The Patient Assignation Number and her initials must be recorded on the Case Report Form.

**8. ABBREVIATIONS**

| ALND | Axillary lymph node dissection |
| --- | --- |
| BD | Blue dye |
| CEUS | Contrast-enhanced ultrasound |
| cN0 | Clinically node negative |
| CNS | Carbon nanoparticle suspensions |
| CRFs | Case Report Forms |
| EBC | Early breast cancer |
| FNR | False negative rates |
| ICBN | Intercostobrachial nerve |
| ICG | Indocyanine green |
| ITT | Intent-to-treat population |
| LTV | Lateral thoracic tributary of the axillary vein |
| NCT | Neoadjuvant chemotherapy |
| NIR | Near-infrared |
| pCR | Pathological complete response |
| RI | Radioisotope |
| SLN | Sentinel lymph nodes |
| SLNB | Sentinel lymph node biopsy |
| SPIO | Superparamagnetic iron oxide |
| TAD | Targeted axillae resection |
| US | Ultrasonography |

**9. REFERENCES**

1. Caudle AS, Cupp JA, Kuerer HM. Management of axillary disease. Surg Oncol Clin N Am 2014; 23: 473-86.
2. Krag DN, Anderson SJ, Julian TB, et al. Technical outcomes of sentinel-lymph-node resection and conventional axillary-lymph-node dissection in patients with clinically node-negative breast cancer: results from the NSABP B-32 randomised phase III trial. Lancet Oncol 2007; 8: 881-88.
3. Krag DN, Anderson SJ, Julian TB, et al. Sentinel-lymph-node resection compared with conventional axillary-lymph-node dissection in clinically node-negative patients with breast cancer: overall survival findings from the NSABP B-32 randomised phase 3 trial. Lancet Oncol 2010; 11: 927-33.
4. Fisher B, Jeong JH, Anderson S, Bryant J, Fisher ER, Wolmark N. Twenty-five-year follow-up of a randomized trial comparing radical mastectomy, total mastectomy, and total mastectomy followed by irradiation. N Engl J Med 2002; 347: 567-75.
5. Krag DN, Weaver DL, Alex JC, Fairbank JT. Surgical resection and radiolocalization of the sentinel lymph node in breast cancer using a gamma probe. Surg Oncol 1993; 2: 335-39; discussion 40.
6. Giuliano AE, Kirgan DM, Guenther JM, Morton DL. Lymphatic mapping and sentinel lymphadenectomy for breast cancer. Ann Surg 1994; 220: 391-401
7. Petrek JA, Senie RT, Peters M, Rosen PP. Lymphedema in a cohort of breast carcinoma survivors 20 years after diagnosis. Cancer 2001; 92: 1368-77.
8. Lucci A, McCall LM, Beitsch PD, et al. Surgical complications associated with sentinel lymph node dissection (SLND) plus axillary lymph node dissection compared with SLND alone in the American College of Surgeons Oncology Group Trial Z0011. J Clin Oncol 2007; 25: 3657-63.
9. McLaughlin SA, Wright MJ, Morris KT, et al. Prevalence of lymphedema in women with breast cancer 5 years after sentinel lymph node biopsy or axillary dissection: objective measurements. J Clin Oncol 2008; 26: 5213-19.
10. McLaughlin SA, Wright MJ, Morris KT, et al. Prevalence of lymphedema in women with breast cancer 5 years after sentinel lymph node biopsy or axillary dissection: patient perceptions and precautionary behaviors. J Clin Oncol 2008; 26: 5220-26.
11. Giuliano AE, Hunt KK, Ballman KV, et al. Axillary dissection vs no axillary dissection in women with invasive breast cancer and sentinel node metastasis: a randomized clinical trial. JAMA 2011; 305: 569-75.
12. Galimberti V, Cole BF, Zurrida S, et al. Axillary dissection versus no axillary dissection in patients with sentinel-node micrometastases (IBCSG 23-01): a phase 3 randomised controlled trial. Lancet Oncol 2013; 14: 297-305.
13. Thevarajah S, Huston TL, Simmons RM. A comparison of the adverse reactions associated with isosulfan blue versus methylene blue dye in sentinel lymph node biopsy for breast cancer. Am J Surg 2005; 189: 236-39.
14. Ferrucci M, Franceschini G, Douek M. New techniques for sentinel node biopsy in breast cancer. Transl Cancer Res. 2018;7 (Suppl 3): S405–17.
15. Giuliano AE, Kirgan DM, Guenther JM, Morton DL. Lymphatic mapping and sentinel lymphadenectomy for breast cancer. Ann Surg 1994; 220: 391-401.
16. Krag D, Weaver D, Ashikaga T, et al. The sentinel node in breast cancer--a multicenter validation study. N Engl J Med 1998; 339: 941-46.
17. Montgomery LL, Thorne AC, Van Zee KJ, et al. Isosulfan blue dye reactions during sentinel lymph node mapping for breast cancer. Anesth Analg 2002; 95: 385-88
18. Gumus M, Gumus H, Jones SE, Jones PA, Sever AR, Weeks J. How long will I be blue? Prolonged skin staining following sentinel lymph node biopsy using intradermal patent blue dye. Breast Care (Basel) 2013; 8: 199-202.
19. Peek MC, Kovacs T, Baker R, Hamed H, Kothari A, Douek M. Is blue dye still required during sentinel lymph node biopsy for breast cancer? Ecancermedicalscience 2016; 10: 674.
20. Bronskill MJ. Radiation dose estimates for interstitial radiocolloid lymphoscintigraphy. Semin Nucl Med 1983; 13: 20-25.
21. Stratmann SL, McCarty TM, Kuhn JA. Radiation safety with breast sentinel node biopsy. Am J Surg 1999; 178: 454-57.
22. Green CH. Technetium-99m production issues in the United Kingdom. J Med Phys 2012; 37: 66-71.
23. Ionising Radiation (Medical Exposure) Regulations (IR(ME)R) | Care Quality Commission. Care Quality Commission. Available at: <https://www.cqc.org.uk/guidance-providers/ionising-radiation/ionising-radiation-medical-exposure-regulations-irmer>. Accessed 2 Dec 2019.
24. Alvarado MD, Mittendorf EA, Teshome M, et al. SentimagIC: A Non-inferiority Trial Comparing Superparamagnetic Iron Oxide Versus Technetium-99m and Blue Dye in the Detection of Axillary Sentinel Nodes in Patients with Early-Stage Breast Cancer. Ann Surg Oncol 2019; 26: 3510-16.
25. Douek M, Klaase J, Monypenny I, et al. Sentinel node biopsy using a magnetic tracer versus standard technique: the SentiMAG Multicentre Trial. Ann Surg Oncol 2014; 21: 1237-45.
26. Ballardini B, Santoro L, Sangalli C, et al. The indocyanine green method is equivalent to the ^99m^Tc-labeled radiotracer method for identifying the sentinel node in breast cancer: a concordance and validation study. Eur J Surg Oncol 2013; 39: 1332-36.
27. He K, Chi C, Kou D, et al. Comparison between the indocyanine green fluorescence and blue dye methods for sentinel lymph node biopsy using novel fluorescence image-guided resection equipment in different types of hospitals. Transl Res 2016; 178: 74-80.
28. Mieog JS, Troyan SL, Hutteman M, et al. Toward optimization of imaging system and lymphatic tracer for near-infrared fluorescent sentinel lymph node mapping in breast cancer. Ann Surg Oncol 2011; 18: 2483-91.
29. Pitsinis V, Provenzano E, Kaklamanis L, Wishart GC, Benson JR. Indocyanine green fluorescence mapping for sentinel lymph node biopsy in early breast cancer. Surg Oncol 2015; 24: 375-79.
30. Polom K, Murawa D, Nowaczyk P, Rho YS, Murawa P. Breast cancer sentinel lymph node mapping using near infrared guided indocyanine green and indocyanine green--human serum albumin in comparison with gamma emitting radioactive colloid tracer. Eur J Surg Oncol 2012; 38: 137-42.
31. Samorani D, Fogacci T, Panzini I, et al. The use of indocyanine green to detect sentinel nodes in breast cancer: a prospective study. Eur J Surg Oncol 2015; 41: 64-70.
32. Somashekhar SP, Kumar CR, Ashwin KR, et al. Can Low-cost Indo Cyanine Green Florescence Technique for Sentinel Lymph Node Biopsy Replace Dual Dye (Radio-colloid and Blue Dye) Technique in Early Breast Cancer: A Prospective Two-arm Comparative Study. Clin Breast Cancer 2020; 20: e576-e83.
33. Sorrentino L, Sartani A, Pietropaolo G, et al. A Novel Indocyanine Green Fluorescence-Guided Video-Assisted Technique for Sentinel Node Biopsy in Breast Cancer. World J Surg 2018; 42: 2815-24.
34. Valente SA, Al-Hilli Z, Radford DM, Yanda C, Tu C, Grobmyer SR. Near Infrared Fluorescent Lymph Node Mapping with Indocyanine Green in Breast Cancer Patients: A Prospective Trial. J Am Coll Surg 2019; 228: 672-78.
35. van der Vorst JR, Schaafsma BE, Verbeek FP, et al. Randomized comparison of near-infrared fluorescence imaging using indocyanine green and 99(m) technetium with or without patent blue for the sentinel lymph node procedure in breast cancer patients. Ann Surg Oncol 2012; 19: 4104-11.
36. Ahmed M, Purushotham AD, Douek M. Novel techniques for sentinel lymph node biopsy in breast cancer: a systematic review. Lancet Oncol 2014; 15: e351-62.
37. Siso C, de Torres J, Esgueva-Colmenarejo A, et al. Intraoperative Ultrasound-Guided Excision of Axillary Clip in Patients with Node-Positive Breast Cancer Treated with Neoadjuvant Therapy (ILINA Trial) : A New Tool to Guide the Excision of the Clipped Node After Neoadjuvant Treatment. Ann Surg Oncol 2018; 25: 784-91.
38. Dominici LS, Negron Gonzalez VM, Buzdar AU, et al. Cytologically proven axillary lymph node metastases are eradicated in patients receiving preoperative chemotherapy with concurrent trastuzumab for HER2-positive breast cancer. Cancer 2010; 116: 2884-89.
39. Kuerer HM, Sahin AA, Hunt KK, et al. Incidence and impact of documented eradication of breast cancer axillary lymph node metastases before surgery in patients treated with neoadjuvant chemotherapy. Ann Surg 1999; 230: 72-78.
40. Li J, Chen X, Qi M, Li Y. Sentinel lymph node biopsy mapped with methylene blue dye alone in patients with breast cancer: A systematic review and meta-analysis. PLoS One 2018; 13: e0204364.
41. Caudle AS, Yang WT, Krishnamurthy S, et al. Improved Axillary Evaluation Following Neoadjuvant Therapy for Patients With Node-Positive Breast Cancer Using Selective Evaluation of Clipped Nodes: Implementation of Targeted Axillary Dissection. J Clin Oncol 2016; 34: 1072-78.
42. Boughey JC, Ballman KV, Le-Petross HT, et al. Identification and Resection of Clipped Node Decreases the False-negative Rate of Sentinel Lymph Node Surgery in Patients Presenting With Node-positive Breast Cancer (T0-T4, N1-N2) Who Receive Neoadjuvant Chemotherapy: Results From ACOSOG Z1071 (Alliance). Ann Surg 2016; 263: 802-07.
43. Donker M, Straver ME, Wesseling J, et al. Marking axillary lymph nodes with radioactive iodine seeds for axillary staging after neoadjuvant systemic treatment in breast cancer patients: the MARI procedure. Ann Surg 2015; 261: 378-82.
44. Caudle AS, Yang WT, Krishnamurthy S, et al. Improved Axillary Evaluation Following Neoadjuvant Therapy for Patients With Node-Positive Breast Cancer Using Selective Evaluation of Clipped Nodes: Implementation of Targeted Axillary Dissection. J Clin Oncol 2016; 34: 1072-78.
45. Siso C, de Torres J, Esgueva-Colmenarejo A, et al. Intraoperative Ultrasound-Guided Excision of Axillary Clip in Patients with Node-Positive Breast Cancer Treated with Neoadjuvant Therapy (ILINA Trial) : A New Tool to Guide the Excision of the Clipped Node After Neoadjuvant Treatment. Ann Surg Oncol 2018; 25: 784-91.
46. Sever A, Jones S, Cox K, Weeks J, Mills P, Jones P. Preoperative localization of sentinel lymph nodes using intradermal microbubbles and contrast-enhanced ultrasonography in patients with breast cancer. Br J Surg 2009; 96: 1295-99.
47. Sever AR, Mills P, Jones SE, et al. Preoperative sentinel node identification with ultrasound using microbubbles in patients with breast cancer. AJR Am J Roentgenol 2011; 196: 251-56.
48. Cox K, Sever A, Jones S, et al. Validation of a technique using microbubbles and contrast enhanced ultrasound (CEUS) to biopsy sentinel lymph nodes (SLN) in pre-operative breast cancer patients with a normal grey-scale axillary ultrasound. Eur J Surg Oncol 2013; 39: 760-65.
49. Harnan SE, Cooper KL, Meng Y, et al. Magnetic resonance for assessment of axillary lymph node status in early breast cancer: a systematic review and meta-analysis. Eur J Surg Oncol 2011; 37: 928-36.
50. Meng Y, Ward S, Cooper K, Harnan S, Wyld L. Cost-effectiveness of MRI and PET imaging for the evaluation of axillary lymph node metastases in early stage breast cancer. Eur J Surg Oncol 2011; 37: 40-46.
51. Shiozawa M, Lefor AT, Hozumi Y, et al. Sentinel lymph node biopsy in patients with breast cancer using superparamagnetic iron oxide and a magnetometer. Breast Cancer 2013; 20: 223-29.
52. Wang AZ, Gu FX, Farokhzad OC. Nanoparticles for cancer diagnosis and therapy. In: Safety of nanoparticles: from manufacturing to medical applications. Webster TG, ed. New York: Springer, 2009: 209–36.
53. Nimura H, Narimiya N, Mitsumori N, Yamazaki Y, Yanaga K, Urashima M. Infrared ray electronic endoscopy combined with indocyanine green injection for detection of sentinel nodes of patients with gastric cancer. Br J Surg 2004; 91: 575-79.
54. Miyashiro I, Miyoshi N, Hiratsuka M, et al. Detection of sentinel node in gastric cancer surgery by indocyanine green fluorescence imaging: comparison with infrared imaging. Ann Surg Oncol 2008; 15: 1640-43.
55. Kusano M, Tajima Y, Yamazaki K, Kato M, Watanabe M, Miwa M. Sentinel node mapping guided by indocyanine green fluorescence imaging: a new method for sentinel node navigation surgery in gastrointestinal cancer. Dig Surg 2008; 25: 103-08.
56. Fujiwara M, Mizukami T, Suzuki A, Fukamizu H. Sentinel lymph node detection in skin cancer patients using real-time fluorescence navigation with indocyanine green: preliminary experience. J Plast Reconstr Aesthet Surg 2009; 62: e373-78.
57. Tsujino Y, Mizumoto K, Matsuzaka Y, Niihara H, Morita E. Fluorescence navigation with indocyanine green for detecting sentinel nodes in extramammary Paget's disease and squamous cell carcinoma. J Dermatol 2009; 36: 90-94.
58. Gioux S, Choi HS, Frangioni JV. Image-guided surgery using invisible near-infrared light: fundamentals of clinical translation. Mol Imaging 2010; 9: 237-55.
59. Tong M, Guo W, Gao W. Use of Fluorescence Imaging in Combination with Patent Blue Dye versus Patent Blue Dye Alone in Sentinel Lymph Node Biopsy in Breast Cancer. J Breast Cancer 2014; 17: 250-55.
60. Chi C, Ye J, Ding H, et al. Use of indocyanine green for detecting the sentinel lymph node in breast cancer patients: from preclinical evaluation to clinical validation. PLoS One 2013; 8: e83927.
61. Wishart GC, Loh SW, Jones L, Benson JR. A feasibility study (ICG-10) of indocyanine green (ICG) fluorescence mapping for sentinel lymph node detection in early breast cancer. Eur J Surg Oncol 2012; 38: 651-56.
62. Sugie T, Sawada T, Tagaya N, et al. Comparison of the indocyanine green fluorescence and blue dye methods in detection of sentinel lymph nodes in early-stage breast cancer. Ann Surg Oncol 2013; 20: 2213-18.
63. Ballardini B, Santoro L, Sangalli C, et al. The indocyanine green method is equivalent to the (9)(9)mTc-labeled radiotracer method for identifying the sentinel node in breast cancer: a concordance and validation study. Eur J Surg Oncol 2013; 39: 1332-36.
64. Xie P, Yang ST, He T, Yang S, Tang XH. Bioaccumulation and Toxicity of Carbon Nanoparticles Suspension Injection in Intravenously Exposed Mice. Int J Mol Sci 2017; 18.
65. Wang LY, Li JH, Zhou X, Zheng QC, Cheng X. Clinical application of carbon nanoparticles in curative resection for colorectal carcinoma. Onco Targets Ther 2017; 10: 5585-89.
66. Li Z, Ao S, Bu Z, et al. Clinical study of harvesting lymph nodes with carbon nanoparticles in advanced gastric cancer: a prospective randomized trial. World J Surg Oncol 2016; 14: 88.
67. Zhao WJ, Luo H, Zhou YM, Gou ZH, Wang B, Zhu JQ. Preoperative ultrasound-guided carbon nanoparticles localization for metastatic lymph nodes in papillary thyroid carcinoma during reoperation: A retrospective cohort study. Medicine (Baltimore) 2017; 96: e6285.
68. Zhang L, Huang Y, Yang C, et al. Application of a carbon nanoparticle suspension for sentinel lymph node mapping in patients with early breast cancer: a retrospective cohort study. World J Surg Oncol 2018; 16: 112.
69. Harlow SP, Krag DN, Ames SE, Weaver DL. Intraoperative ultrasound localization to guide surgical excision of nonpalpable breast carcinoma. J Am Coll Surg 1999; 189: 241-46.
70. Kaufman CS, Jacobson L, Bachman B, Kaufman LB. Intraoperative ultrasonography guidance is accurate and efficient according to results in 100 breast cancer patients. Am J Surg 2003; 186: 378-82.
71. Luini A, Zurrida S, Paganelli G, et al. Comparison of radioguided excision with wire localization of occult breast lesions. Br J Surg 1999; 86: 522-25.
72. Rahusen FD, Taets van Amerongen AH, van Diest PJ, Borgstein PJ, Bleichrodt RP, Meijer S. Ultrasound-guided lumpectomy of nonpalpable breast cancers: A feasibility study looking at the accuracy of obtained margins. J Surg Oncol 1999; 72: 72-76.
73. Rahusen FD, Bremers AJ, Fabry HF, van Amerongen AH, Boom RP, Meijer S. Ultrasound-guided lumpectomy of nonpalpable breast cancer versus wire-guided resection: a randomized clinical trial. Ann Surg Oncol 2002; 9: 994-98.
74. Snider HCJ, Morrison DG. Intraoperative ultrasound localization of nonpalpable breast lesions. Ann Surg Oncol 1999; 6: 308-14.
75. Clough KB, Nasr R, Nos C, Vieira M, Inguenault C, Poulet B. New anatomical classification of the axilla with implications for sentinel node biopsy. Br J Surg 2010; 97: 1659-65.
76. Pavlista D, Eliska O, Duskova M, Zikan M, Cibula D. Localization of the sentinel node of the upper outer breast quadrant in the axillary quadrants. Ann Surg Oncol 2007; 14: 633-37.
77. Suami H, Pan WR, Mann GB, Taylor GI. The lymphatic anatomy of the breast and its implications for sentinel lymph node biopsy: a human cadaver study. Ann Surg Oncol 2008; 15: 863-71.
78. Ivanovic N, Granic M, Randjelovic T, Todorovic S. Fragmentation of axillary fibrofatty tissue during dissection facilitates preservation of the intercostobrachial nerve and the lateral thoracic vein. Breast 2008; 17: 293-95.
79. Mok CW, Tan SM, Zheng Q, Shi L. Network meta-analysis of novel and conventional sentinel lymph node biopsy techniques in breast cancer. BJS Open 2019; 3: 445-52.
