# Supplementary figures and images for: Ultrasound-assisted carbon nanoparticle suspension mapping versus dual tracer-guided sentinel lymph node biopsy in patients with early breast cancer (ultraCars): phase III randomized clinical trial
Source: Br J Surg. 2022 Sep 8;109(12):1232–8. doi: 10.1093/bjs/znac311 (PMC10364740; doi:10.1093/bjs/znac311)

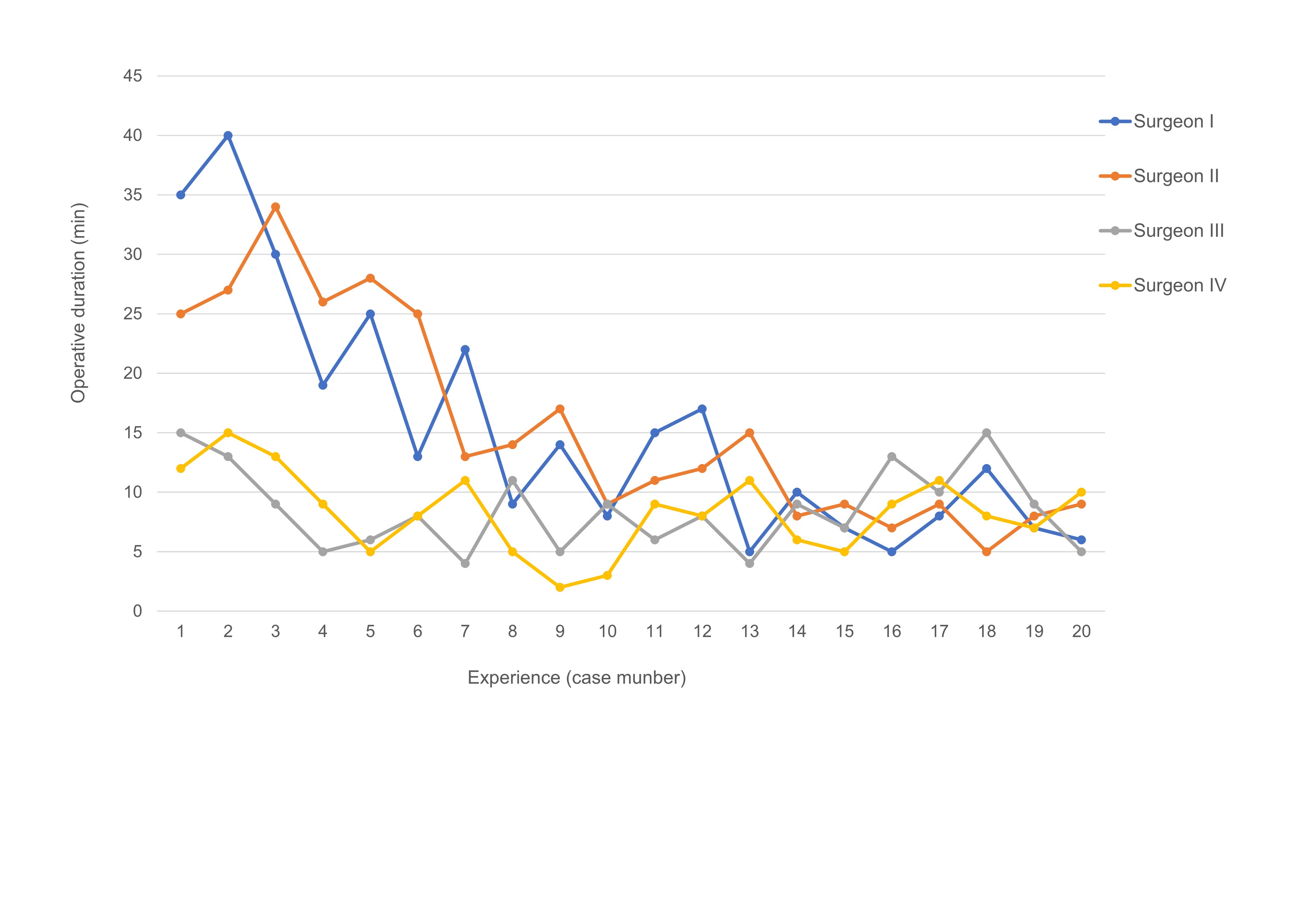

Supplement: znac311_Supplementary_Data [file znac311_supplementary_data.zip › Supplementary_Figure_1.jpg]
